# Complete Genome Sequence of *Mycobacterium bovis* Clinical Strain 1595, Isolated from the Laryngopharyngeal Lymph Node of South Korean Cattle

**DOI:** 10.1128/genomeA.01124-15

**Published:** 2015-10-01

**Authors:** Narae Kim, Yunho Jang, Jin Kyoung Kim, Soyoon Ryoo, Ka Hee Kwon, Shin Seok Kang, Hyeon Seop Byeon, Hee Soo Lee, Young-Hee Lim, Jae-Myung Kim

**Affiliations:** a Bacterial Disease Division, Animal and Plant Quarantine Agency, Anyangsi, Gyeonggido, Republic of Korea; b Department of Integrated Biomedical and Life Science, College of Health Science, Korea University, Seoul, Republic of Korea; c Chungcheongbukdo Veterinary Service, Jecheonsi, Chungcheongbukdo, Republic of Korea

## Abstract

*Mycobacterium bovis* strain 1595 was isolated from the lymph node of South Korean native cattle. The complete genome sequence of strain 1595 was determined in 2 contigs and was found to be 4,351,712 bp in size, with a 65.64% G+C content and 4,358 predicted protein-coding genes.

## GENOME ANNOUNCEMENT

*Mycobacterium bovis* is responsible for bovine tuberculosis (BTB), an OIE (World Organization for Animal Health) notifiable disease, and can cause a chronic infectious disease in livestock, wildlife, and humans (1, 2). BTB is a contagious disease that can be caused by intake of raw milk or inhalation of droplets from infected cattle (3). To confirm the presence of this disease and its transmission in various populations, it is necessary to completely understand the metabolism and mechanisms of pathogenicity of *M. bovis* strains by sequencing and annotation to compare with other strains. In particular, we are concerned about the *M. bovis* strains in South Korea. Our objective was to introduce the complete genome sequence of *M. bovis* clinical strain 1595, isolated from the laryngopharyngeal lymph node of BTB-infected cattle (*Bos taurus* coreanae) in South Korea.

The complete genome of *M. bovis* clinical strain 1595 was constructed *de novo* using Illumina and Pacific Biosciences (PacBio) sequencing data. Sequencing analysis was performed in Chunlab, Inc., and the National Instrumentation Center for Environmental Management (NICEM). The genome filtering, assembly, and reassembly were performed using PacBio SMRT Analysis version 2.3.0 and RS HGAP Assembly.2 (4). Raw reads (889,283,194-bp total) were filtered to remove single-molecule real-time (SMRT) bell adapters and short (<500-bp) and low-quality (80% accuracy) reads. There were 2 contigs and a total length of 4,352,971 bp with 136.25× coverage. Illumina- and PacBio-assembled contigs were combined and processed to generate a complete genome sequence. Subsequently, the completed circular sequence was amended by mapping with Illumina data.

Consed (http://www.phrap.org/consed/consed.html) was used for editing the sequence and CLC bio was used for mapping and correction. Overlapping regions were trimmed and final mapped reads were 6,828,614 (99.99%) with 1,126,855,424 bp and 258.95× coverage to a genome size of 4.31 Mb. Gene prediction was carried out using Glimmer version 3 (5), and the results were annotated by comparing with the NCBI nonredundant (NR) database (6). The tRNAs and rRNAs were identified using tRNAscan-SE version 1.21 (7) and RNAmmer version 1.2 (8), respectively. By use of the BLASTP program, each gene was identified and annotated based on similarities. Finally, by use of Artemis version 1.4 (https://www.sanger.ac.uk/resources/software/artemis/), the annotated open reading frames (ORFs) were edited or corrected (9).

The final circularized genome of *M. bovis* 1595 was 4,351,712 bases with a G+C content of 65.64%. A total of 4,358 protein-coding sequences (CDSs), 1 rRNA operon, and 45 tRNAs were predicted. Phylogenetic analysis based on complete genome sequences from the NCBI microbial sequence databases was constructed using Clustal W (10), and the phylogenetic distances were closer (0.05), suggesting sequence similarity and similar evolution of genes. We expect that this genome sequence will provide valuable information for understanding the disparity in the virulence and epidemiological traits among *M. bovis* genotypes.

### Nucleotide sequence accession number.

The assembly and annotation files of *M. bovis* clinical strain 1595 were deposited in GenBank under the accession number CP012095.
